# Vascular Air Embolism During Bronchoscopy Procedures- Incidence, Pathophysiology, Diagnosis, Management and Outcomes

**DOI:** 10.7759/cureus.1087

**Published:** 2017-03-09

**Authors:** Venkatkiran Kanchustambham, Swetha Saladi, Kris Mehta, John Mwangi, Zafar Jamkhana, Setu Patolia

**Affiliations:** 1 Pulmonary and Critical Care Medicine, Saint Louis University School of Medicine; 2 Internal Medicine, Saint Louis University School of Medicine; 3 Pulmonary and Critical Care Medicine , Saint Louis University School of Medicine

**Keywords:** argon plasma coagulation(apc), nd-yag laser, bronchoscopy, air embolism, venous air embolism, arterial air embolism, end tidal co2, paradoxical embolism, patent foramen ovale

## Abstract

Vascular air embolism (VAE) is a rare, but potentially fatal complication of invasive medical or surgical procedures. It is a very rare complication of bronchoscopy and is most frequently reported with therapeutic bronchoscopy with Argon plasma coagulation (APC) or neodymium-doped yttrium aluminum garnet (Nd-YAG) laser. Despite being rare, as a result of its high chance of mortality and morbidity, it is imperative that physicians have high clinical suspicion to allow for early recognition and treatment. In this article, we provide a concise review of the incidence, pathophysiology, diagnosis management and outcomes of air embolism during bronchoscopy procedures.

## Introduction and background

Vascular air embolism (VAE) is a rare, preventable, but serious complication arising from iatrogenic invasive procedures resulting in significant morbidity and mortality [1-2]. This rare complication has been traditionally associated with surgeries like the cardiopulmonary bypass, hip replacement, and craniotomy performed with the patient in sitting position. However, it is increasingly recognized as a complication in a wide variety of invasive procedures like endoscopy, angiography, central/peripheral venous access and tissue biopsy [3]. The estimated incidence of this rare complication is one in 772 per one series, while another study showed the incidence of iatrogenic embolism to be 2.65 per 100,000 hospitalizations. In the latter study, the majority (30%) of the iatrogenic air embolism cases were due to endovascular procedures [3].

Flexible bronchoscopy is a relatively safe procedure done for evaluation and management of respiratory disease [4]. The reported mortality with diagnostic bronchoscopy is around 0.012%, and morbidity ranged between 0.14% and 2.5% per one series [5]. With the advent of formalized fellowship training programs in interventional pulmonology in the United States, many of the advanced diagnostic and therapeutic procedures that were performed only at tertiary and specialist centers in the past are rapidly becoming standard practice and are widely available at many centers now [6]. Argon plasma coagulation (APC) and Nd-YAG laser therapy are the therapeutic bronchoscopic techniques that have been associated with air embolism [6]. Due to its rarity and with the only recent increase in the number of these procedures, there needs to be high clinical suspicion to allow for early recognition and treatment.

Schlaepfer K, et al. [7],  first documented the incidence of air embolism following various diagnostic and therapeutic procedure on the lung in 1922. Cerebral air embolism was reported following diagnostic bronchoscopy by Wherrett, et al. [8]. Systemic gas embolism has been reported as a complication with the bronchoscopic use of neodymium-doped yttrium aluminum garnet (Nd-YAG) laser in the airways [9-14]. In the study by Nicholas P Lang, et al. [10], of 62 patients who have undergone Nd-YAG laser treatment for endobronchial carcinoma, eight patients manifested perioperative cardiac or cerebral events which were thought to be due to air embolism. Tellides G, et al. [14], reported two cases of gas embolism causing cardiac and neurologic complications after bronchoscopic Nd-YAG laser tumor ablation.

Bronchoscopic argon plasma coagulation (APC) resulting in systemic gas embolism has been reported as a complication with resultant cardiovascular collapse and cerebral embolism [15,18]. Reddy, et al. [15], reported three cases of intracardiac gas embolism (two fatal) occurring after APC over three years. Goldman, et al. [16], reported a case of cardiac arrest due to left ventricular gas embolism after bronchoscopic APC. Yasmeen, et al. [17], reported a case of cerebral gas embolism from bronchoscopic APC.In the study by Reichle, et al. [18], three patients developed post-procedural neurological complications and two patients died (myocardial infarction, hypovolemic shock). The neurological and cardiac complications in these five patients may have been due to intracardiac gas embolism.

Hence in this article, we set out to review the pathophysiology, clinical signs and symptoms, treatment, and prognosis of vascular air embolism during bronchoscopy.

## Review

Vascular air embolism (VAE) is the entry of gas into the vascular structures which can result in serious mortality and morbidity. VAE is classified based on the mechanism of entry and the ultimate site they lodge at (venous and arterial gas embolism). 

A venous air embolism occurs when the air enters the systemic venous system and reaches the lungs via the pulmonary arteries leading to trapping of air bubbles in the pulmonary capillary bed. This phenomenon results in decreased gas exchange, cardiac arrhythmia, pulmonary hypertension, right ventricular strain and cardiac failure. 

Systemic arterial embolism manifests due to the entry of the air into the pulmonary veins or directly into the arteries of the systemic circulation [1,3]. The introduction of gas into the aorta causes the entry of gas bubbles into nearly all organs. The air emboli in the vessels of the skeletal muscles or viscera are well tolerated, but embolization to the cerebral or coronary circulation may result in severe morbidity or death because of the vulnerability of the heart and brain to short periods of hypoxia. These emboli cause pathologic changes by two mechanisms: a reduction in perfusion distal to the obstruction and an inflammatory response to the presence of air bubble [19-20]. 

The exact mechanisms responsible for the development of systemic air embolism during bronchoscopy procedures resulting in cardio-cerebrovascular complications are not known. Some mechanisms have been proposed to explain the vascular air embolism in the bronchoscopic use of Nd-YAG laser and was extrapolated to explain the possible mechanism of vascular air embolism in APC [14]. 

A broncho-vascular fistula is an abnormal communication formed between the pulmonary arteries/veins and airways due to heat coagulation and mechanical destruction of the tumor and surrounding tissue by the endobronchial application of APC and laser. The gasses exiting the tip of the APC, Nd-YAG laser probe, or occlusion of the proximal recanalized bronchus by the bronchoscope can generate high pressures in airways. This high pressure can result in the air being pushed from the airways into the pulmonary arteries/veins via the broncho-vascular fistula [14]. Studies showed a positive correlation between the rate of gas flow and the presence of gas emboli after thermal ablation therapy in the airways [21]. Endobronchial application of the APC and laser in the trachea is associated more commonly with gas embolization to the right atrium, presumably via systemic veins, whereas ablation in bronchi is associated more often with gas embolization to the left atrium through the pulmonary venous system [15].

Paradoxical embolization occurs when gas migrates from the venous system into the arterial system. Possible mechanisms include an intracardiac right-to-left shunt (patent foramen ovale), arteriovenous malformations in the lungs or overwhelming of the pulmonary capillary filter mechanism [14,22].

During or after therapeutic bronchoscopy procedures, a delayed recovery from general anesthesia or cardiovascular instability indicates the possible occurrence of vascular air embolism [1-2]. Air embolism can present with and variable symptoms and signs of cardiac arrest, stroke and chest pain, paresthesia, convulsions, paralysis, nausea, visual disturbances and headache [3]. The overall mortality rate reported for all causes of iatrogenic air embolism was 21% as per one study with mortality reaching > 50% in patients with cardiac arrest as initial presentation [23]. As there are only handful of case reports of air embolism during bronchoscopy, it is hard to comment on the mortality associated with air embolism in bronchoscopy. However, overall mortality and morbidity related to therapeutic bronchoscopy from all complications is 0.61% and < five percent respectively per one series [5].

Numerous real-time monitors are used for the detection of air embolism during surgical procedures that carry a high-risk of air embolisms such as neurosurgical procedures or cardiac surgery. These include; transesophageal echocardiography (TEE) and precordial doppler which can visualize intravenous and intracardiac air bubbles [24-25]. However, TEE use in bronchoscopy is limited by the fact that its invasive, requires expertise, constant monitoring and is operator dependent [24]. Precordial Doppler is a non-invasive monitoring method that is very sensitive in detecting smallest amounts of air in the heart and may be a useful tool for detecting air embolism during bronchoscopy. The precordial doppler does, however, have limitations. It may be difficult to place the probe on the chest in obese patients, The Doppler is overly sensitive and does not differentiate between a clinically significant and insignificant embolism causing false alarms.The Doppler does not function during electrocautery because of radio frequency interference and is unable to detect air embolism during that time [25]. Hence these devices may not be useful for continuous monitoring during bronchoscopy but can be used to confirm if there is a suspicion of air embolism [24,26].

All patients during bronchoscopy have heart rate, respiratory rate, blood pressure and oxygen saturation monitored continuously as per the guidelines [6,27]. As many of the bronchoscopy procedures are done under conscious sedation, end-tidal carbon dioxide which measures the exhaled carbon dioxide is not routinely monitored [6]. The presence of air in the pulmonary vessels interferes with gaseous exchange and increases the dead space resulting in retention of carbon dioxide. The retention of carbon dioxide results in rapid fall in exhaled carbon dioxide that is detected by the end tidal carbon dioxide monitor which may be an early sign of air embolism. The change in end-tidal carbon dioxide, however, is non-specific and is seen in other conditions such as hyperventilation, low cardiac output, other types of emboli, and chronic obstructive pulmonary disease (COPD). The wide availability, low- cost and noninvasive nature of the monitor makes it an ideal choice to monitor for air embolism during therapeutic bronchoscopy procedures to allow for early recognition and treatment [28,30].

In cases of cerebral air embolism, the intracerebral air on computed tomography (CT) of the head will appear as negative density streaks reproducing a cast of the cerebral arteries or as bilateral, multiple well-defined low-density areas with cerebral edema or infarction [31]. However, cerebral air embolism cannot be definitively ruled out in the presence of normal imaging studies [32]. Hence, clinical evaluation is still preferred for the assessment of cerebral air embolism. CT chest may show small amounts of air on the chest, but this can be seen even in patients with clinically insignificant air [3]. 

Hemodynamic support is the first mainstay in the acute treatment of gas embolism. Cardiac arrhythmias are common, and cardiopulmonary resuscitation and catecholamine administration for hemodynamic support may be necessary. Airway protection with endotracheal intubation is essential for the treatment of hypoxia and adequate oxygen delivery. These patients should be placed on 100% oxygen. High flow oxygen has been established as a treatment to reduce air bubble size and aid the reabsorption of nitrogen gas from the bubble into the blood. Volume expansion with intravenous fluids is not only essential for resuscitation but also to avoid a wide pressure gradient which can increase gas embolism risk [1,3,16-17,33].

In the case of venous air embolism, Durant's maneuver is performed, by placing the patient in the left lateral decubitus position and Trendelenburg position. This maneuver allows the air bubble to move out of the right ventricular outflow tract (RVOT) and into the right atrium, thus relieving the obstruction of the pulmonary vasculature responsible for the cardiopulmonary collapse. Physical evacuation of gas emboli with chest compressions can improve forward blood flow even without hemodynamic compromise [3].

The patient should be transferred to intensive care unit (ICU) for careful monitoring and management and be evaluated for advanced interventions that include hyperbaric oxygen therapy, air aspiration and extracorporeal membrane oxygenation (ECMO). Hyperbaric oxygen therapy involves a patient breathing 100% oxygen at a higher pressure than atmospheric sea level and is indicated in the presence of neurological deficits [34].

## Conclusions

In conclusion, physicians should be aware of the risk of air embolism during or after bronchoscopic procedures in patients who show cardiopulmonary instability and neurologic symptoms. Even though this is a rare complication, we believe that the rate of this complication can be reduced during bronchoscopy if the following is kept in mind; keep the flow rate at the tip of the bronchoscope to the lowest possible, avoid advancing the bronchoscope so that it does not completely occlude the bronchus and utilize non-contact technique when using the APC. Have a high index of suspicion when there is a delayed recovery from general anesthesia or a transitional stage of impaired consciousness after the procedure. Consider continuous end-tidal carbon dioxide monitoring during the therapeutic bronchoscopy to detect even small decrements in exhaled carbon dioxide which may be an early clue to the possibility of vascular air embolism.
